# The benefits of socioemotional learning strategies and video formats for older digital immigrants learning a novel smartphone application

**DOI:** 10.3389/fragi.2024.1416139

**Published:** 2024-06-24

**Authors:** Jaclyn H. Ford, Ryan T. Daley, Elizabeth A. Kensinger

**Affiliations:** ^1^ Department of Psychology and Neuroscience, Boston College, Chestnut Hill, MA, United States; ^2^ Department of Psychology, Gordon College, Ken Olsen Science Center, Wenham, MA, United States

**Keywords:** learning strategies, memory, technology, older learners, socioemotional processing, learning format

## Abstract

The need to continually learn and adjust to new technology can be an arduous demand, particularly for older adults who did not grow up with digital technology (“older digital immigrants” or ODIs). This study tests the efficacy of socioemotional learning strategies (i.e., encoding information in a socially- or emotionally-meaningful way) for ODIs learning a new software application from an instructional video (*Experiment 1*) or a written manual (*Experiment 2*). An experiment-by-condition effect was identified, where memory was greatest for participants engaging socioemotional learning strategies while learning from a video, suggesting a synergistic effect of these manipulations. These findings serve as a first step toward identifying and implementing an optimal learning context for ODIs to learn new technologies in everyday life.

## Introduction

Navigating the modern world requires us to constantly learn, update, and adjust to new technology. This need to learn new technology can present challenges for middle-aged and older adults who did not grow up with technology as part of their daily experience. Here we refer to this group of individuals as “older digital immigrants” (ODI). Unlike “digital natives”, or individuals who grew up using technology such as smartphones, laptops, tablets, etc., ODIs were introduced to these technologies in mid-to-later life. Although technology can ultimately be helpful to ODIs–allowing them to keep track of everything from their own daily schedules to the medication regimens of those for whom they may be assuming a caregiver role -- this technology is constantly changing, requiring them to update their memories of how to interact with their electronic devices, even after learning to use them. For ODIs, the demands of this constantly shifting technological landscape can be particularly challenging, drawing on the types of memory abilities with which individuals of their age show particular difficulty (see Park and Festini, 2017), and can have a significant impact on their daily lives (Parikh et al., 2016).

One factor contributing to older adults’ difficulty learning new technology may relate to their tendency to utilize poor learning strategies (e.g., Old and Naveh-Benjamin, 2012). Although older adults report *knowing* a number of effective strategies for improving memory (Hache et al., 2018), they are still likely to rely heavily on less effective strategies (Aronov et al., 2015). Providing older adults with strategies to learn information can boost their memory, but these strategies are often effortful to use and difficult to generalize beyond laboratory settings. For instance, if asked to learn word pairs, older adults might be instructed to form a sentence linking these words together (Frankenmolen et al., 2017; Kuhlmann and Touron, 2017). Although older adults *can* learn to use these strategies, because they are effortful to use, they have been most effectively engaged by older adults with high executive function or IQ (e.g., Bender and Raz, 2012; Frankenmolen et al., 2017), and they tend not to be spontaneously generalized to other learning tasks that older adults encounter. As a result, the examination of learning strategies that may generalize to individuals beyond those with high executive function or IQ is needed in order to assist the growing older adult population in the context of the changing technological landscape.

The current research examines the efficacy of memory strategies that capitalize on older adults’ known strengths. Both young and older adults are more likely to remember content that elicits an emotional response (e.g., Kensinger, 2009) or is encoded using a *self-referencing* mnemonic strategy (Gutchess et al., 2010; Gutchess et al., 2007). In the current study, we consider these processes together as *socioemotional strategies*, based on evidence of shared mechanisms supporting episodic memory (see Gutchess and Kensinger, 2018 for review). Indeed, extensive research has revealed that there are age-related gains in socioemotional abilities, with older adults giving high priority to the implementation of these processes (Carstensen et al., 1999; Charles and Carstensen, 2010; Scheibe and Carstensen, 2010). Prior research suggests that it is possible for older adults to assist their memory performance by connecting the content they are being asked to learn to these prioritized socioemotional goals (e.g., Carstensen and Turk-Charles, 1994; Fung and Carstensen, 2003; Kensinger and Gutchess, 2017).

The current study tests the efficacy of a socioemotional encoding strategy as a learning tool that can be taught to ODIs and deployed flexibly to enable them to effectively learn to utilize new technologies. There are several reasons to believe socioemotional encoding strategies would be effective, not only relative to baseline (i.e., the absence of an instructed learning strategy) but also relative to standard learning strategies. First, traditional learning strategies that benefit younger adults (e.g., repetition, generating a sentence from novel information, generating a mental image of novel information) have failed to help older adults as much (e.g., Fox et al., 2016), likely because they relied heavily on self-initiated control processes that are impaired in older adults (e.g., Dunlosky and Hertzog, 1998). Indeed, prior research suggests that *learner-centric* interventions, such as the socioemotional instructions used in the current study, seem to show the most success for older adult learners (Bottiroli et al., 2013; Flegal and Lustig, 2016). Second, the use of socioemotional strategies appears to require less effort than the use of other types of strategies and may be more automatically employed by older adults in memory tasks (Kensinger and Gutchess, 2017). Finally, socioemotional strategies may help older adults feel less threatened by new technology. Older adults often reject new technology, sometimes before even attempting to learn how to use it (Nilsson and Townsend, 2010; Czaja et al., 2013). Older adults’ frustration with technology can come from many sources (Van Volkom et al., 2014; Hill et al., 2015), but often it is because they do not see how the technology would be beneficial to daily life (Murthy and Mani, 2013; Pew Research Center, 2014), or they are frustrated or overwhelmed by the associated learning demands (Larsson et al., 2013). Despite this initial reaction, once they learn the new technology, older adults often report the same degree of benefit from technology adoption as younger adults (Pew Research Center, 2014; Chopik, 2016; Li et al., 2024). Socioemotional learning strategies may assist older adults’ learning as well as their ability to more quickly understand how the technology may be beneficial to their daily life.

In addition to individual strategies, encoding may also be influenced by the mode of presentation. In the current study, we focus on the difference between a written manual, and a guided video tutorial. On one hand, video presentation, particularly in unsupervised online studies, could allow the participant to “zone out” (i.e., not engage meaningfully with the video) or to let the video run while directing mental resources to another task. This attention lapse could result in reduced encoding of new material (Baddeley et al., 1984), where the additional effort of reading the written manual may ensure deeper encoding (Craik and Byrd, 1982). Indeed, many prior studies conducted in young adults have shown superior memory for content in news reports presented in *print* relative to audiovisual presentation (e.g., Gunter et al., 1984; Furnham and Gunter, 1985; Gunter et al., 1986; Furnham and Gunter, 1987; DeFleur et al., 1992).

On the other hand, the benefit of print over video may be specific to videos in which there is limited redundancy between visual and verbal information, such as those typically used in news reports. When videos with greater visual/verbal overlap are used (such as news videos designed for children), young adults show no benefit of print or video (Furnham et al., 2002) or a benefit of video presentation (Walma van der Molen and van der Voort, 2000). The video in the current study walked participants through each step, both verbally and visually, and therefore likely contained sufficient overlap to confer a benefit over text. Presenting verbal and visual information can also reduce cognitive load during encoding, facilitating transfer of information from working to long-term memory (Mayer, 2017), Finally, an instructional video may encourage learners to engage with the material by capturing their attention. We are naturally attracted to moving objects (Howard and Holcombe, 2010), allowing videos to capture our attention and maintain it for longer durations of time. Videos may also capture attention by being more socially-relevant than written manuals, often including the voices and/or faces of those providing instruction. This more socially-relevant format may encourage us to treat the new material as more social or emotional, leading to a baseline socioemotional memory benefit (see Kensigner and Gutchess, 2017).

In this current study, we compare the efficacy of instruction for a socioemotional learning strategy to a control condition (no instructed learning strategy) and to instruction for a standard learning strategy on the ability of adults ages 55+ (those who are not “digital natives” and would not have had access to personal computing until they were adults) to learn a new software application. We do so using two different types of learning materials: an instructional video that includes the social context of a human voice (Expt 1) and a PDF manual devoid of overt social cues (Expt 2). Across these two experiments, we test the hypothesis that, for those who are older digital immigrants, socioemotional learning strategies can be engaged to make detailed information, like the functions within a new software application, more memorable compared to when they are learned with no strategies or standard strategies.

## Experiment 1

### Methods

#### Participants

Data from Experiment 1 are from 234 participants (age 55–78, *M* = 62.85, *SD* = 5.08; 153 females) who reported being a native English speaker age 55+ and who completed both parts of a 2-day online experiment with adequate performance on attention and quality assurance checks. Participants were recruited from Amazon’s Mechanical Turk (MTurk) and from a database of participants who had expressed interest in completing studies in our lab. The current study focuses on participants ages 55 and older, as it is likely that these participants did not grow up using digital technology and had to acquire that skill later in life (i.e., “older digital immigrants”, or ODIs). Although all participants were able to sign-up for and complete an online study, and 99.6%, reported using their computer daily, we wanted to limit our sample to participants who did not have professional computer experience. Therefore, we excluded participants who reported specialized computer training (31 participants) as a computer programmer, app developer, or computer science teacher. Therefore, the final sample for the study included 203 participants (ages 55–76; *M* = 62.91, *SD* = 4.94)^1^. Participants were compensated $11 for participating in the two-part study and were consented in accordance with the requirements of the Institutional Review Board at Boston College.

Participants were randomly assigned to one of three groups at the beginning of the study: Control (*n* = 70, ages 55–75, *M* = 62.46, *SD* = 4.48), Standard Strategy Manipulation (*n* = 65, ages 56–75, *M* = 63.65, *SD* = 5.42) and Socioemotional Strategy Manipulation (*n* = 68, ages 55–76, *M* = 62.68, *SD* = 4.91; see *Procedure* section below for description of the three groups). This sample provided 89.6% power to detect a medium effect across groups (f = .25). The conditions did not differ in age of participant (*F* (2,200) = 1.09, *p* = .337, η_p_^2^ = .011) or sex (*Χ* ^*2*^ (2) = 1.73, *p* = .422).

#### Procedure

The current study took place over the course of two half-hour study sessions, both conducted online using Qualtrics and Amazon’s Mechanical Turk. On Day 1, participants were randomly assigned into one of three experimental conditions: Control (*n* = 70), Standard Strategy (*n* = 65), and Socioemotional Strategy (*n* = 68). All data and materials, including the videos used, have been made publicly available at the OSFHome and can be accessed at DOI 10.17605/OSF.IO/TFWSY (https://osf.io/tfwsy/?view_only=29bd4af188934f07a68e81a8344738a5).

**FIGURE 1:**
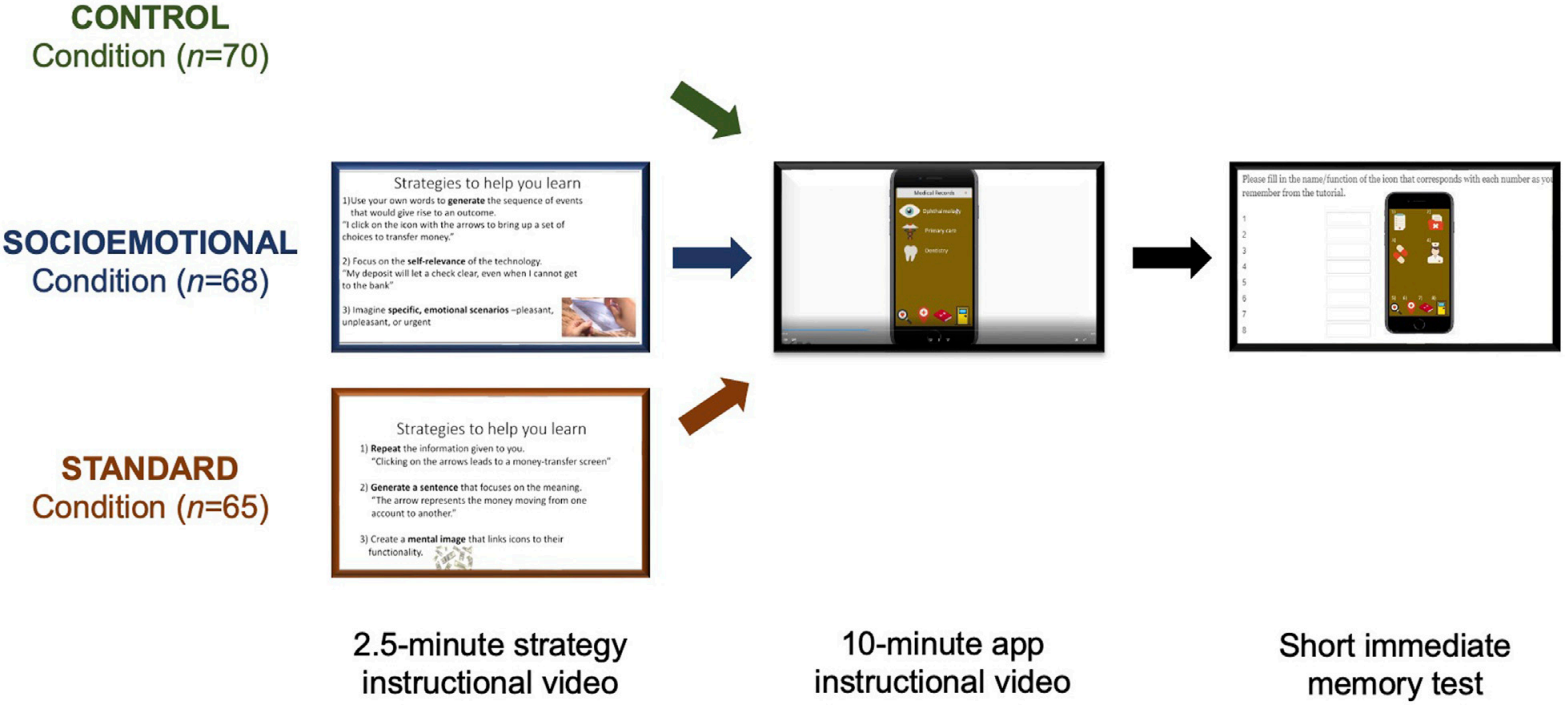
Visual depiction of the Experiment 1 encoding procedure for the Control (*n* = 70), Socioemotional (*n* = 68), and Standard (*n* = 65) learning conditions. All participants viewed a 10-min instructional video—teaching them the functions of a novel smartphone application—immediately followed by a short memory test. Prior to the application instructional video, participants in the Socioemotional and Standard learning conditions viewed a 2.5-min video instructing them on how to use the socioemotional or standard strategies, respectively.

Day 1: *Encoding* (See Figure 1 for depiction of Day 1 procedures).

Participants in the Control condition were presented with a 10-min video that introduced the functions of a new smartphone application. This mock application (designed for the purposes of this study, only, to ensure that no participant had prior familiarity with it) was a medical application that helped users to manage medical data and contact medical professionals. In the 10-min video, a female narrator (author E.A.K.) walked participants through a number of possible uses for the application and the function of all icons. Although the narrator’s voice could be heard, they were not seen on the screen. The video was followed by a brief memory test in which participants were presented with an image of the home page of the app and asked to label the 8 icons.

The instructional video and immediate memory test used in the Standard and Socioemotional Strategy conditions were identical to those in the Control condition. However, prior to viewing this video, participants in the strategy conditions watched additional video tutorials on specific learning strategies. The “Standard Strategy” manipulation condition was designed to mimic the deep encoding strategies known to generally benefit memory and that have most commonly been used to try to enhance memory performance (e.g., Kirchhoff et al., 2012). Participants in the Standard Strategy condition viewed a 2.5 min video teaching them to:

- Repeat novel information
- Generate a sentence from novel information
- Create a mental image of novel information

The “Socioemotional Strategy” manipulation condition was designed to rely on processes that are typically relatively preserved in older adults, including self-referential processing and emotional engagement. Participants in the Socioemotional Strategy condition viewed a 2.5 min video teaching them to:

- Generate actions related to novel information
- Engage in the self-relevance of novel information
- Focus on the emotion related to novel information

After watching the video tutorials for these strategies, participants watched three additional 1–2 min videos that walked them through a practice of each strategy. After each strategy video, participants were asked if they understood how to use the strategy and were given the opportunity to practice it again if not. Importantly, while the instructional video was presented by a female narrator, using conversational tone and inflection, the videos used to train participants on strategy use were narrated by a computer with an emotionless tone and with stable prosody, using Amazon Polly text-to-speech (https://aws.amazon.com/polly/). This was done because, while the same instructional video was used in all conditions, the training videos differed across conditions. We wanted to ensure that it was only the *content* of those trainings that differed, with no differences in prosodic or other vocal social cues.

After completing all tutorials and practices, participants in the strategy conditions were presented with the 10-min instructional video walking them through the medical application. As in the Control condition, participants in these conditions completed a brief identification memory test immediately after the video. The encoding survey took approximately 20–30 min for participants to complete and can be found at DOI 10.17605/OSF.IO/TFWSY.

Day 2: Retrieval

On Day 2, approximately 24-h after memory encoding, all participants completed the same retrieval task, regardless of encoding condition. The memory task included free response, multiple-choice, and matching questions. The full retrieval survey can be found at DOI 10.17605/OSF.IO/TFWSY.

*Scenarios*: First, participants were asked to describe 3 distinct scenarios where they could use the application. For each, they were asked to include:

1) A description of the situation
2) An explanation for why this application would be helpful
3) A step-by-step description of what they would do in the app
4) A description of the appearance and location of icons

Responses could earn up to 1 point each for the description of the situation, explanation of why the app would be helpful, and the description of the icons, and up to 2 points for the description of steps to be taken to accomplish the goal, for up to 5-points per response.

*Free response*: Participants were also asked to respond to four more specific free response questions; for these questions, the number of possible points (listed below) was based on the total number of pieces of information needed to fully respond to the prompt:

1) Describe how they would use the app to find an existing medical report (up to 3 points)
2) Describe the medical contacts page (up to 8 points)
3) Describe how to add particular medical records (up to 3 points)
4) Describe the prescriptions page and how to use it (up to 4 points)

Free responses were scored by two researchers who were blind to encoding condition. To establish inter-rater reliability, the seven free response questions for a subset of 35 participants were scored by both raters. Scores were highly reliable (average Cronbach’s alpha = .97, with alphas ranging from .88 to .99 across questions) and inconsistent responses were discussed to establish agreement. The responses from the remaining participants were divided up between raters.

*Multiple-choice:* Participants were presented with 9 multiple-choice questions in which they were shown a page from the app and asked to identify the icon that would be used to perform a particular function. These were scored as either correct or incorrect, with correct responses earning 1 point.

*Matching*: Participants were presented with 15 hypothetical scenarios and asked to match each with the appropriate icon from the home screen. These were scored as either correct or incorrect, with correct responses earning 1 point.

The retrieval task took participants approximately 15–25 min to complete, followed by a 5-min survey that asked participants to consider their engagement in the task. Participants were asked how motivated they were to learn the new application and whether they would learn and use a similar application if it were available.

They were then asked to consider what strategies they used during the task. Participants were provided with a list of 15 possible strategies and were asked to rate, on a 1-5 scale, the extent to which they employed each during the encoding task. They then were asked to rank all 15 strategies in order of most to least used. These strategies could be categorized as “standard”, “socioemotional”, or “other”; “other” referred to general strategies that many people use when learning new information (memorize the function, spending time learning the function, paying and keeping attention). These strategies can be found in the full survey (DOI 10.17605/OSF.IO/TFWSY), but are also listed in Supplementary Material. Finally, participants completed a series of attention and quality assurance questions to establish inclusion eligibility (see survey at DOI 10.17605/OSF.IO/TFWSY).

#### Data analysis

##### Memory Score

Participants’ memory scores were calculated by adding up their points across all memory tasks and dividing by the total possible score so that scores ranged from 0 to 1. The first 32 participants (12 control, 12 socioemotional, and 8 standard) were not presented with all questions. Seventeen were missing two of the free responses questions while 15 were only presented with the initial encoding questions and the matching task. Therefore, their total possible score was not the same as the remaining participants. Scores for these participants were calculated by dividing their score by an adjusted “total possible” score (i.e., the total score possible based on the questions that they were presented) to take these differences into account. All analyses were conducted using the full sample, but follow-up analyses confirmed that findings were consistent when only including the 202 participants who answered all memory questions.

##### Analyses

A factorial ANOVA was used to examine the effects of condition (control, standard, or socioemotional) on memory performance. To examine self-reported use of different strategies, the average ratings of “socioemotional”, “standard”, and “other” strategies were calculated. A mixed-model ANOVA was used to examine strategy use with condition (control, standard, or socioemotional) as a between subject factor and strategy type (socioemotional, standard, and other) as a within-subject factor on self-reported strategy use. A final factorial ANCOVA looked at the effects of condition (between subject factor) and strategy use (other, standard, and socioemotional strategy use, as covariates) on memory performance. This analysis looked at the main effects of each strategy as well as the interactions with condition.

### Results

**FIGURE 2:**
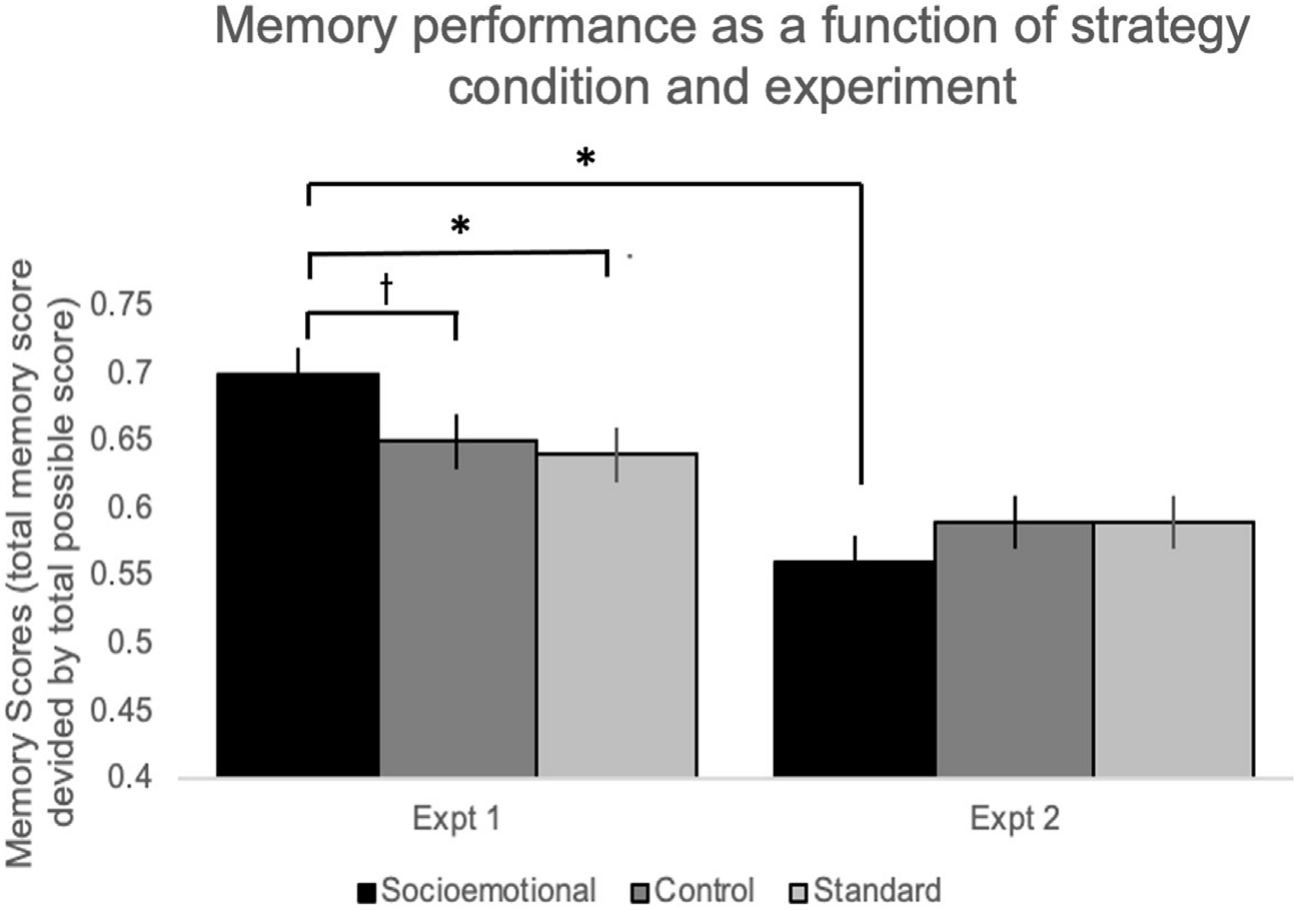
Average memory score as a function of strategy training condition (Control, Socioemotional, or Standard) and Experiment (Experiment 1: video or Experiment 2: written). * = *p* < .05, t = *p* < .1.

There was a significant main effect of condition on memory (*F* (2,200) = 3.38, *p* = .036, η_p_^2^ = .033), driven by significantly greater memory in the socioemotional strategy condition (*M* = .70, *SE* = .02) relative to control (*M* = .65, *SE* = .02; *p* = .040, LSD correction) and standard (*M* = .64, *SE* = .02; *p* = .017, LSD correction; See Figure 2).

**FIGURE 3:**
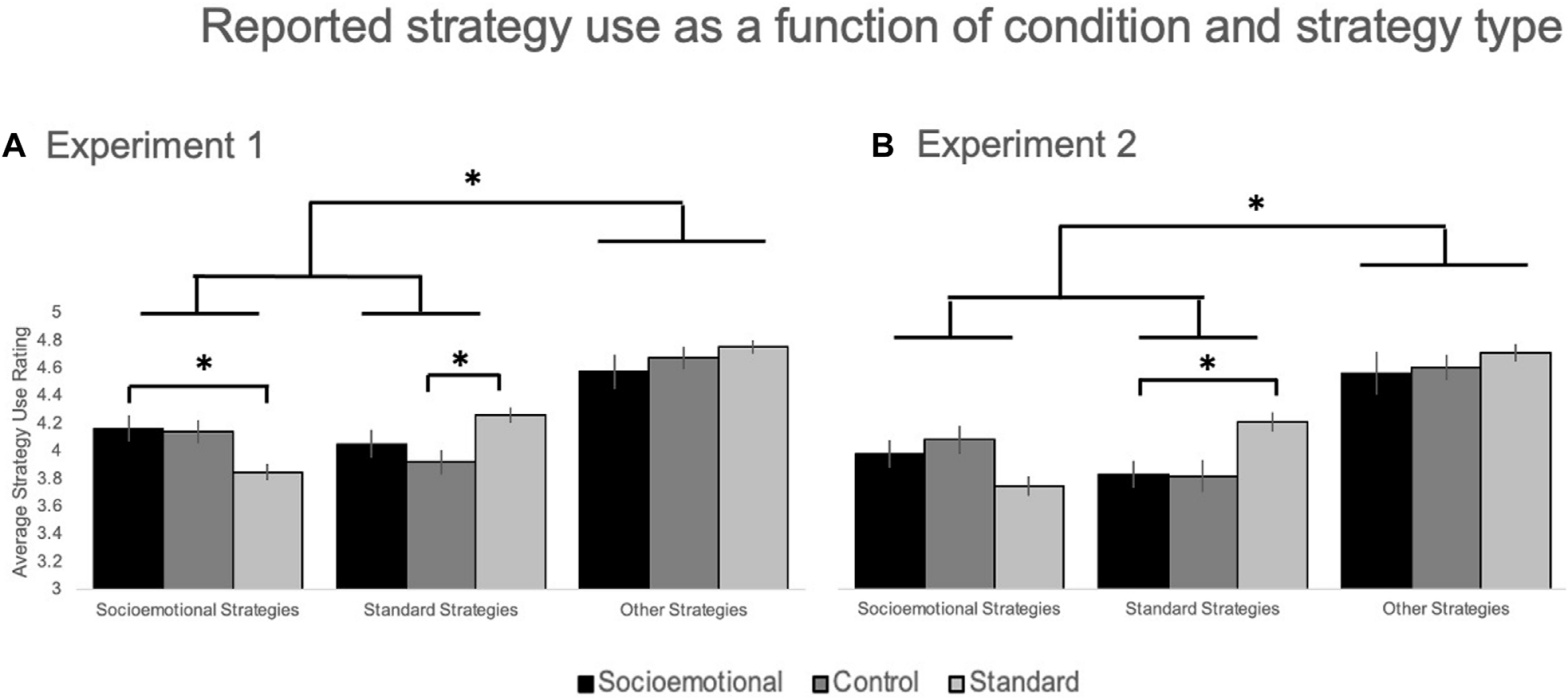
Average reported strategy use as a function of strategy type (socioemotional, standard, or other), strategy training condition (Control, Socioemotional, or Standard), and Experiment (**A**: Experiment 1: video or **B**: Experiment 2: written). * = *p* < .05.

There was a main effect of strategy use (*F* (2,400) = 102.28, *p* < .001, *η*_*p*_^*2*^ *=* .34); participants reported using other strategies (*M* = 4.67, *SE* = .03) to a greater extent than socioemotional strategies (*M* = 4.05, *SE* = .06) or standard strategies (*M* = 4.08, *SE =* .05). There was no main effect of condition (*F* (2,200) = .09, *p* = .911, *η*_*p*_^*2*^ = .001), but there was a significant condition-by-strategy interaction (*F* (4,400) = 9.29, *p* < .001, *η*_*p*_^*2*^ *=* .085; See Figure 3A). This interaction was driven by significantly greater *standard* strategy in the standard condition (*M* = 4.26, *SE* = .09) relative to the control condition (*M* = 3.92, *SE* = .09; *p* = .006, LSD correction) and significantly greater endorsements of *socioemotional* strategy use in the socioemotional condition (*M* = 4.17, *SE* = .11)) relative to the standard condition (*M* = 3.85, *SE* = .11; *p* = .038, LSD correction).

The ANCOVA examining the relation between self-reported strategy use and memory performance revealed no significant effects (condition: *F* (2,191) = .28, *p* = .755, *η*_*p*_^*2*^ *=* .003; standard strategy use: *F* (1,191) = .19, *p* = .660, *η*_*p*_^*2*^ *=* .001; socioemotional strategy use: *F* (1,191) = .14, *p* = .705, *η*_*p*_^*2*^ *=* .001; other strategy use: *F* (1,191) = .90, *p* = .345, *η*_*p*_^*2*^ *=* .005; condition-by-standard: *F* (2,191) = .01, *p* = .990, *η*_*p*_^*2*^ *<*.001; condition-by-socioemotional: *F* (2,191) = .90, *p* = .409, *η*_*p*_^*2*^ *=* .009; condition-by-other: *F* (2,191) = .09, *p* = .918, *η*_*p*_^*2*^ *=* .001).

#### Summary

Being trained on socioemotional strategies prior to encoding led to significantly improved memory performance compared to training on standard memory strategies.

In terms of self-reported strategy use, participants in the standard memory condition reported using standard strategies to a greater extent than those in the control condition. Participants in the socioemotional condition reported using significantly more socioemotional strategies compared to those in the standard strategy condition; however, they did not differ from those in the control condition in their reported strategy use. One possibility is that the method of information delivery, a video in which a human voice explained different functions of the app, caused participants in the control condition to naturally gravitate toward more socioemotional strategies, even without explicit instruction. Experiment 2 used an information source void of social context (a PDF manual) to explore whether this format would alter the efficacy of socioemotional strategies for learning the novel medical app. In addition, comparing Experiment 1 and 2 allows us to evaluate the overall efficacy of video vs. written manuals.

## Experiment 2

### Methods

#### Participants

197 participants (age 55–76, *M* = 62.19, *SD* = 4.66; 129 females), who all reported being native English speakers, completed both parts of a 2-day online experiment and passed attention and quality-control checks. Participants were recruited from Amazon’s Mechanical Turk (MTurk), Prolific, and from a database of participants who had expressed interest in completing studies in our lab. No participants from Experiment 1 were allowed to participate in Experiment 2. As in Experiment 1, although most participants reported daily use of technology (96%), we wanted to limit our sample to participants who did not have professional computer experience and excluded the 56 participants who reported specialized computer training. Therefore, the final sample for the study included 141 participants (ages 55–76; *M* = 62.70, *SD* = 4.73; see Supplementary Material for analyses including all participants). Participants were compensated $11 for participating in the two-part study and were consented in accordance with the requirements of the Institutional Review Board at Boston College.

As with Experiment 1, participants were randomly sorted into one of three groups at the beginning of the study: Control (*n* = 55, ages 55–76, *M* = 62.83, *SD* = 4.92), Standard Strategy Manipulation (*n* = 39, ages 56–71, *M* = 62.87, *SD* = 4.19), Socioemotional Strategy Manipulation (*n* = 49, ages 55–76, *M* = 61.43, *SD* = 5.00). The conditions did not differ as a function of age (*F* (2,138) = .13, *p* = .883, η_p_^2^ = .002) or sex (*Χ* ^*2*^ (2) = 4.14, *p* = .126).

While Experiment 1 was entirely conducted prior to the onset of the COVID-19 pandemic in North America, Experiment 2 spanned multiple phases. It began before the pandemic (*n* = 61), but an additional 34 participants were collected during the initial phase of the pandemic (March-August 2020). Because we became concerned that factors related to stay-at-home orders might influence results, data collection was paused until September-October 2021, at which point vaccinations were widely available and most pandemic-related restrictions had been lifted (*n* = 46). Effects of timing, relative to the pandemic, are reported in Supplementary Material.

#### Procedure

**FIGURE 4:**
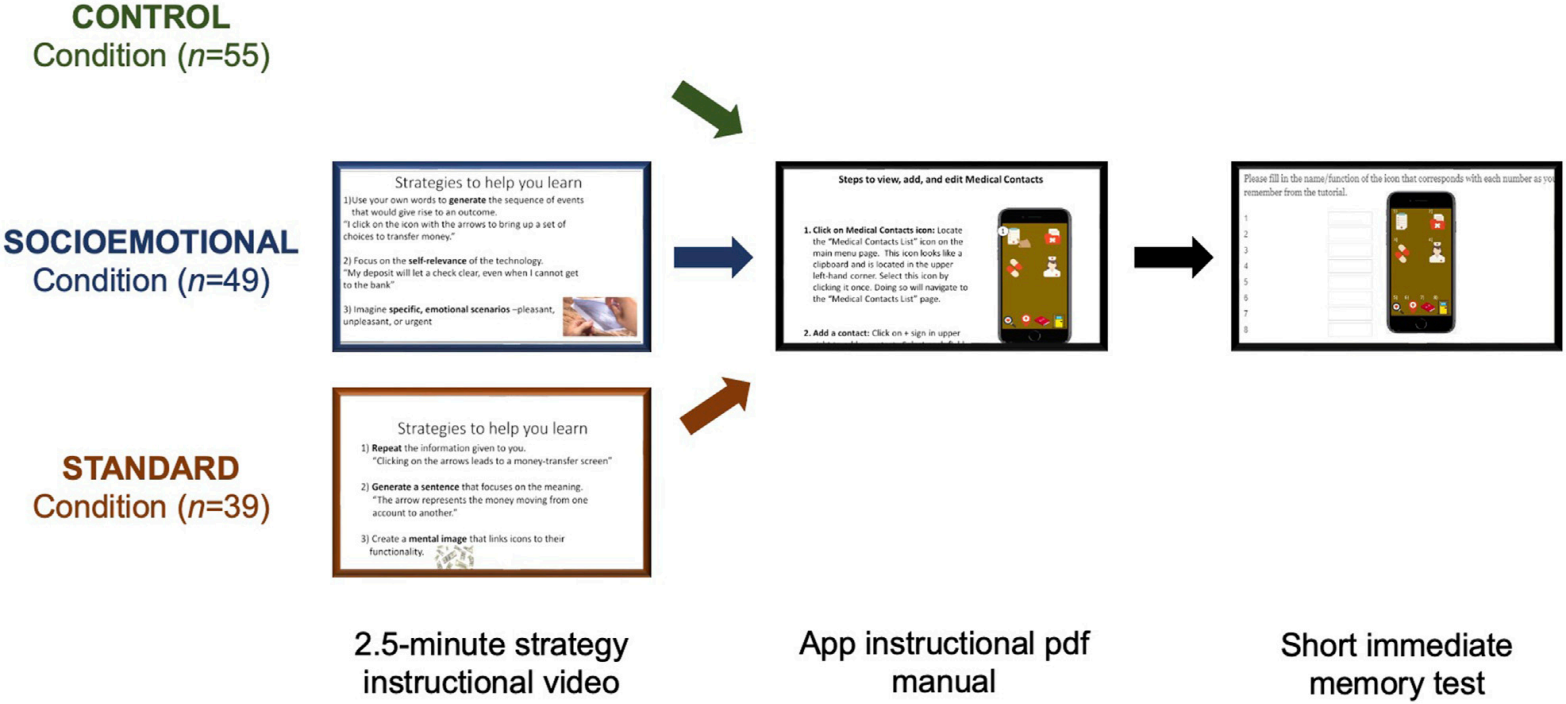
Visual depiction of the Experiment 2 encoding procedure for the Control (*n* = 55), Socioemotional (*n* = 49), and Standard (*n* = 39) learning conditions. All participants were given 10 minutes to read through a written manual that provided information about the new smartphone application. This was immediately followed by a short memory test. Prior to the application instructional video, participants in the Socioemotional and Standard learning conditions viewed a 2.5-min video instructing them on how to use the socioemotional or standard strategies, respectively.

Experiment 2 was identical to Experiment 1, but instead of viewing a 10-min instructional video introducing the functions of a new smartphone application, participants were given 10 min to read through an 8-page pdf manual describing these functions (manual can be found at DOI 10.17605/OSF.IO/TFWSY). Participants were not allowed to advance from the manual until the time limit was up. Strategy tutorial videos and all memory questions remained the same as in Experiment 1 (See Figure 4).

#### Data analysis

Data scoring and analysis were conducted in the same way as they were for Experiment 1. In addition, exploratory analyses compared memory and strategy use across Experiments 1 and 2 to determine whether information presentation (video v. pdf) influenced performance or the effects of the socioemotional manipulation. To maximize consistency in methodology across experiments, these analyses only include those participants in Experiment 1 who completed all memory questions.

### Results

Unlike the Experiment 1 results, memory performance did not differ as a function of strategy condition (*F* (1,138) = .79, *p* = .457, *η*_*p*_^*2*^ = .011; Figure 2).

There was a main effect of strategy use (*F* (2,276) = 99.46, *p* < .001, η_p_^2^ = .419), where participants reported using other strategies (*M* = 4.63, *SE* = .04) to a greater extent than socioemotional strategies (*M* = 3.94, *SE* = .07) or standard strategies (*M* = 3.95, *SE =* .06). There was no main effect of condition (*F* (2,138) = .34, *p* = .715, *η*_*p*_^*2*^ = .005), but there was a significant condition-by-strategy interaction (*F* (4,276) = 7.71, *p* < .001, *η*_*p*_^*2*^ *=* .101; See Figure 3B). This interaction was driven by a significant effect of condition on standard strategy use (*F* (2,138) = 3.89, *p* = .023, η_p_^2^ = .053), where standard strategy use was significantly greater in the standard condition (*M* = 4.21, *SE* = .12) relative to the control (*M* = 3.82, *SE* = .10; *p* = .013, LSD correction) and socioemotional conditions (*M* = 3.83, *SE* = .11; *p* = .017, LSD correction).

The ANCOVA examining the relation between self-reported strategy use and memory performance revealed a significant main effect of “other” strategy use (*F* (1,129) = 4.21, *p* = .042, *η*_*p*_^*2*^ *=* .025), with greater memory in individuals who endorsed greater use of “other” strategies (*r* = .37, *p* < .001). No other effects were significant (condition: *F* (2,129) = 1.67, *p* = .193, *η*_*p*_^*2*^ *=* .025; standard strategy use: *F* (1,129) = 1.47, *p* = .227, *η*_*p*_^*2*^ *=* .011; socioemotional strategy use: *F* (1,129) = .01, *p* = .912, *η*_*p*_^*2*^ *<*.001; condition-by-standard: *F* (2,129) = .44, *p* = .647, *η*_*p*_^*2*^ *=* .007; condition-by-socioemotional: *F* (2,129) = .62, *p* = .539, *η*_*p*_^*2*^ *=* .010; condition-by-other: *F* (2,129) = .53, *p* = .589, *η*_*p*_^*2*^ *=* .008).

#### Comparison of experiments 1 and 2

There was a significant effect of experiment on memory performance (*F* (2,338) = 33.46, *p* < .001, η_p_^2^ = .090), where memory performance was significantly greater in Experiment 1: video (*M* = .67, *SE* = .01) compared to Experiment 2: manual (*M* = .58, *SE* = .01). There was no significant effect of condition (*F* (1,338) = .33, *p* = .716, *η*_*p*_^*2*^ = .002), but a significant experiment-by-condition interaction (*F* (2,338) = 3.35, *p* = .036, η_p_^2^ = .019) that reflects the significant effect of condition seen in Experiment 1, but not Experiment 2 (see results reported above).

There were no main effects of experiment (*F* (1,338) = 2.34, *p* = .127, *η*_*p*_^*2*^ = .007) or condition (*F* (2,338) = .33, *p* = .719, *η*_*p*_^*2*^ = .002) on strategy use, and strategy use was not influenced by the interaction of experiment with condition (*F* (2,338) = .14, *p* = .869, *η*_*p*_^*2*^ = .001), strategy (*F* (2,676) = .78, *p* = .458, *η*_*p*_^*2*^ = .002), or strategy and condition (*F* (4,676) = .46, *p* = .765, *η*_*p*_^*2*^ = .003). As was seen in both Experiment 1 and Experiment 2, there was a main effect of strategy use (*F* (2,676) = 196.76, *p* < .001, η_p_^2^ = .368) and a significant condition-by-strategy interaction (*F* (4,676) = 15.975, *p* < .001, *η*_*p*_^*2*^ *=* .086).

#### Summary

Experiment 2 was designed to examine whether effects of condition on memory and strategy use were specific to a video learning format. The significant difference in overall memory between Experiment 1 and 2 suggests that learning format may influence how ODIs learn new technology, with a video generally leading to better memory than a pdf, although the experiment-by-condition interaction suggests that this may only be the case when participants are asked to focus on socioemotional encoding strategies. Similarly, the enhancing effect of socioemotional condition in Experiment 1 was not replicated in Experiment 2, suggesting that there may be a synergistic effect of using videos and socioemotional strategies.

The effects of condition on strategy use in Experiment 2 were the same as in Experiment 1, with no effects of experiment or experiment-by-condition interactions. In other words, although memory was influenced by learning format, this was not mediated by self-reported changes to strategy use.

## Discussion

The current study was the first to examine the effects of socioemotional strategies on older digital immigrants’ learning of a novel smartphone application. Results point to benefits conveyed by learning information in a socioemotional context, but only within the context of a video tutorial. When a training video was used (Experiment 1), there was a significant memory benefit for socioemotional learning strategies. However, this memory benefit did not extend to learning via a manual (Experiment 2). Similarly, the modality effect of video (Experiment 1) relative to pdf (Experiment 2) was specific to the socioemotional strategy condition. Thus, memory performance was best when an instructional video was watched with socioemotional learning strategies engaged (see Figure 2).

These findings suggest actionable changes that ODIs can make in their attempts to more effectively learn new technology. In particular, the results suggest that such individuals could benefit from the use of tutorials or training videos. As they watch these videos, thinking about the self-relevance of each step and of specific, emotional scenarios in which they would execute it, may also provide learning benefits. These strategies may be less demanding for older adults or more intuitive for them to use, which could lead to real-world benefits.

The findings surrounding subjective judgements of strategy use are more difficult to interpret without additional research. Although both experiments revealed that training participants to use specific strategies can lead to subtle changes in the likelihood that they utilize those strategies, these patterns were weaker than expected. Further, there was limited evidence for a link between self-reported strategy use and memory performance. It is possible that the retrospective self-report ratings employed in the current study to evaluate strategy use did not adequately capture real-time use of the strategies. Future studies could innovate ways to include more objective or real-time measures of strategy use. It was notable that the most participants reported using “other” strategies more often than specific learning strategies. This is not surprising as these strategies (e.g., “I kept my attention focused on the task”, see Supplementary Material) were selected to reflect overall intention and effort on the encoding task. There were also fewer of these strategies in the ranking list (4) relative to the list of socioemotional and standard strategies (6 each).

Finally, the finding that ODIs may learn better from a video relative to a written manual is intriguing, as it reflects a simple change that they may make to more effectively learn new technology. Further research will be needed, however, to understand the possible mechanisms underlying this benefit. One possible explanation is that videos, being a more socially-relevant format, naturally encourage socioemotional strategies during encoding. If so, participants may have benefitted from the enhancement conferred upon neutral content encoded in a socioemotional context (see Kensigner and Gutchess, 2017). Videos may also be more engaging than text, maintaining participants’ attention throughout the entire encoding duration with changing visual and auditory content. Indeed, attention is captured by motion (Howard and Holcombe, 2010), abrupt onsets (Yantis and Jonides, 1984), and color singletons (i.e., an item of one color contrasted against a backdrop of other colored items; Pashler, 1988), all of which can be incorporated into videos. The extent to which videos are easier for older adults to attend to and learn from may also depend on literacy engagement, as frequent leisure reading can improve skills such as verbal working memory and episodic memory (Stine-Morrow et al., 2022). This finding suggests that reading for pleasure can have enhancing effects on memory that should be explore further. In addition, future research should consider individual differences in literacy engagement when considering the benefits of video to a written manual.

It is important to note that the current study focused on learning formats where individuals passively receive pre-recorded information, which we believe reflects the way most ODIs learn new applications in their daily lives. It is possible that hands-on training, with the ODI performing application functions on their own, could provide better learning than either of these approaches (i.e., the *enactment effect*; Engelkamp, 1998).

There is an expansive literature focusing on strategies to make educational videos more or less effective (see Brame, 2016 for review). Although the current informational video was designed with some recommendations in mind (e.g., having a concise, clear, and focused message in which audio and visual messages are aligned), there were others that were intentionally not implemented. For instance, the education literature highlights the benefits of social cues such as narrators using a conversational tone or interacting with the viewer (Brame, 2016). The current study kept the narrator’s tone neutral and informative to avoid imbuing the video with additional socioemotional cues, thereby focusing the video v. manual comparison as tightly as possible. Social cues may also differentially affect young and older adults, introducing confounds beyond the scope of the current study. For instance, having an instructor on screen (Wang and Antonenko, 2017) can facilitate learning in undergraduates, but other factors (e.g., whether the instructor is an older adult or a younger adult) could affect the magnitude of benefits in older adults. The current study did not show a narrator on the screen to eliminate this potential confound. Future research should be conducted to see if additional social cues (e.g., faces, gestures, conversational tone, etc.) further support new learning in ODIs.

The current study also focused on the modality of the smartphone app instruction, keeping the training for strategy use constant. An important follow-up would be to explore the potential impact of strategy training modality. Given the significant modality-by-condition interaction identified in the current study, it is possible that ODIs may not benefits from socioemotional strategy instruction that is done in writing.

As with most studies that are conducted online, the current research has some significant limitations that should be considered when interpreting the results. First, although the current study excluded ODIs with specialized computer training, the online nature of the study means that participants had developed a relatively high level of digital competency (i.e., ability to sign up for and complete an online study). It is possible that the results of the current study may not extend to older adults who do not have a high baseline technical ability.

The current research was also affected by the onset of the COVID-19 pandemic halfway through Experiment 2. The onset of the pandemic was associated with increases in anxiety and stress (Rodriguez-Seijas et al., 2020; Morin et al., 2021; Fields et al., 2022) which may have made it more difficult for participants to learn new information. Stay-at-home orders and changes to work-from-home policies may also have influenced the sample of participants available for online research studies or have increased the technological capabilities of ODIs who had no prior experience. To control for these changes, a portion of Experiment 2 was conducted in September-October 2021, when many of these policies had stopped and subjective stress and negative affect had lowered (Fields et al., 2022). Further, exploratory analyses suggest that the differences between Experiment 1 and 2 exist even in those participants from Experiment 2 who completed the study prior to the pandemic (see Supplementary Material).

## Conclusion

The current study serves as a first step in understanding how socioemotional processing and presentation format may aid ODI in learning a new smartphone application. Technology is a central and critical component of the modern world, and one that may provide obstacles to many older adults. The current research suggests that how new technology is learned may assist use of this technology: Memory performance was significantly enhanced when participants learned about the application from an instructional video and utilized socioemotional encoding strategies. Future research will build on these findings to find ways to facilitate the use of these strategies in everyday life.

## Data Availability

The datasets presented in this study can be found in online repositories. The names of the repository/repositories and accession number(s) can be found below: https://osf.io/tfwsy/?view_only&equals;29bd4af188934f07a68e81a8344738a5.

## Footnotes

1 The analysis that includes these computer experts is included in the Supplementary Material.
