# Supplementary material for: The benefits of socioemotional learning strategies and video formats for older digital immigrants learning a novel smartphone application
Source: Front Aging. 2024 Jun 24;5:1416139. doi: 10.3389/fragi.2024.1416139 (PMC11228105; doi:10.3389/fragi.2024.1416139)
Supplement: Supplementary file 1 [file DataSheet1.docx]

**SUPPLEMENTARY MATERIALS**

1. ***Effects of Study and COVID Timing on Memory and Strategy Use***
2. ***Analysis including participants with specialized computer training***
3. ***List of memory strategies***
4. ***Effects of Study and COVID Timing on Memory and Strategy Use***

Although Experiment 1: video was entirely conducted prior to the onset of the COVID-19 pandemic, Experiment 2: manual was conducted in multiple stages. Data collection began before the pandemic, (*n*=61), an additional 34 participants were tested during the initial phase of the pandemic (March-August 2020), and a final 46 participants were tested during Sept-October 2021, when COVID-19 cases were low and many participants were vaccinated. Follow-up analyses were conducted to determine whether these timing differences affected the study results.

First, we conducted the Experiment 1 v. 2 analyses using only the Experiment 2 participants who were tested prior to the pandemic (*N*=61). As in the main analysis, memory was significantly better in Experiment 1: video (*M*=.64, *SE*=.009; *F*(1,232)=5.81, *p*=.02, *η_p_^2^*=.02) than in Experiment 2: manual (*M*=.60, *SE*= .02). The experiment-by-condition interaction showed the same pattern as was seen in the full analysis, but was reduced to a trend (*F*(2,232)=2.85, *p*=.06, *η_p_^2^*=.02), likely due to low power.

In a second set of analyses, we looked at the effect of COVID timing within Experiment 2. There was a trending effect of timing on memory (*F*(2,132)= 2.65, *p*=.08, *η_p_^2^*=.04); memory was better prior to the COVID-19 pandemic (*M*=.60, *SE*=.02) and the initial phase of the pandemic (*M*= .59, *SE*=.02) compared to more than a year later (*M*=.54, *SE*=.02). There was no effect of condition (*F*(2,132)= .87, *p*=.42, *η_p_^2^*=.01) nor a condition-by-timing interaction (*F*(4,132)= .89, *p*=.47, *η_p_^2^*=.03).

As in the primary analysis, there was a main effect of strategy condition on strategy use (*F*(2,264)=96.61, *p*<.001, *η_p_^2^*=.42), qualified by a strategy-by-condition interaction (*F*(4,264)=9.65, *p*<.001, *η_p_^2^*=13). There was no main effect of condition *F*(2,132)=.19, *p*=.83, *η_p_^2^*=.003), a strategy-by-timing interaction (*F*(4,264)=.63, *p*=.64, *η_p_^2^*=.01), or a strategy-by-timing-by-condition interaction (*F*(8,264)=1.89, *p*=.06, *η_p_^2^*=.05). There was a significant effect of study timing (*F*(2,132)=3.46, *p*=.03, *η_p_^2^*=.05), qualified by a significant timing-by-condition interaction (*F*(4,132)=2.70, *p*=.03, *η_p_^2^*=.08). This interaction was driven by a significant effect of timing in the control condition (*F*(2,50)=8.37, *p*=.001, *η_p_^2^*=.25), but not the socioemotional (*F*(2,46)=1.47, *p*=.24, *η_p_^2^*=.06) or standard condition (*F*(2,36)=.64, *p*=.53, *η_p_^2^*=.03). In the control group, participants reported greater overall strategy use before the pandemic (*M*=4.45, *SE*=.11) and immediately after the pandemic (*M*=4.23, *SE*=.18) compared to a year and a half later (*M*=3.79, *SE*=.12).

1. ***Analysis including participants with specialized computer training***

In the primary analysis, participants who reported specialized computer training were excluded in an effort to reduce variability in the sample and focus on our target population: older adults who are relatively new to digital technology. The same analyses are presented below with all participants included.

*Experiment 1*

There was a trending effect of strategy condition on memory (*F*(2,231)=2.68, *p*=.07, *η_p_^2^*=.02) and no effect on reported strategy use (*F*(2,231)=.19, *p*=.83, *η_p_^2^*=.09). As was seen in the main analysis, there was a significant effect of strategy type on strategy use (*F*(2,462)=114.30, *p*<.001, *η_p_^2^*=.33), qualified by a strategy-by-condition interaction (*F*(4,462)=11.57, *p*<.001, *η_p_^2^*=.09).

*Experiment 2*

There was no effect of strategy condition on memory (*F*(2,194)=.18, *p*=.83, *η_p_^2^*=.002) or strategy use (*F*(2,194)=.004, *p*>.99, *η_p_^2^*<.001). As was seen in the main analysis, there was a significant effect of strategy type on strategy use (*F*(2,388)=101.90 *p*<.001, *η_p_^2^*=.34), qualified by a strategy-by-condition interaction (*F*(4,388)=5.30, *p*<.001, *η_p_^2^*=.05).

1. ***List of memory strategies***

The following strategies were presented to participants in a random order. For each statement, participants used a Likert scale ranging from “strongly agree” to “strongly disagree.”

Colors, which were not included on participants’ surveys, are included here to indicate strategy category. Blue strategies are emphasized in “standard” condition and red strategies in “socioemotional” condition.

Strongly disagree…Disagree…Neither Agree nor Disagree…Agree…Strongly Agree

1……………………….2………………………3……………………4…………………….5

I tried to memorize the function of each icon.

I spent time trying to learn the information.

I paid close attention to what was being shown.

I kept my attention focused on the task.

I generated specific instances when I would select an icon.

I thought about the importance of using each icon.

I thought about the usefulness of the application for my day-to-day life.

I thought about pleasant outcomes that could result from using the software application.

I imagined stressful situations that could be made less stressful by using the application.

I imagined specific times when I would use the software application.

I thought about what each icon looks like.

I repeated information after it was presented.

I thought about the meaning behind each graphic.

I thought about the general design of the software.

I rehearsed the content represented by each icon.

I rehearsed the content contained within the software application.
